# Supplementary material for: Magnaporthe oryzae effector MoSPAB1 directly activates rice Bsr-d1 expression to facilitate pathogenesis
Source: Nat Commun. 2023 Dec 18;14:8399. doi: 10.1038/s41467-023-44197-9 (PMC10728069; doi:10.1038/s41467-023-44197-9)
Supplement: Supplementary file 1 — Supplementary Information [file 41467_2023_44197_MOESM1_ESM.pdf]

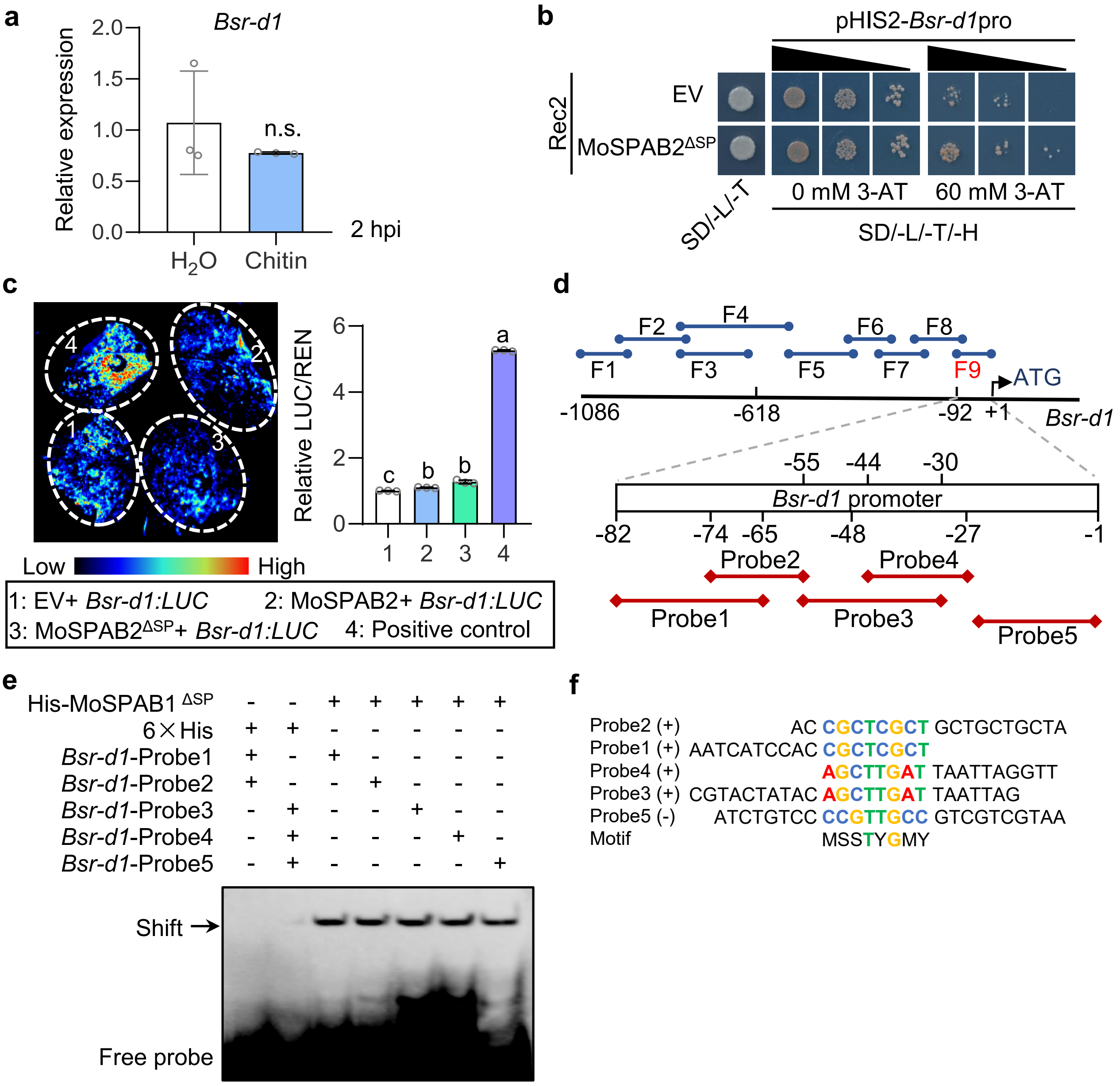

Supplementary Fig. 1 Identification of upstream regulators of *Bsr-d1* derived from *M. oryzae*.

a Expression level of *Bsr-d1* 2 hr post chitin treatment. *OsUbq5* was used as an internal control. Values are mean ± SD, n=3 biologically independent samples (Student's two-sided *t*-test, n.s. indicates no significance). Source data are provided as a Source Data file.

b Determination of MoSPAB2$^{\Delta SP}$ binding to the *Bsr-d1* promoter in a yeast one-hybrid assay. The *Bsr-d1* promoter was fused to the HIS2 reporter and MoSPAB2$^{\Delta SP}$ to GAL4 AD. Yeast cells transformed with the reporter and an effector construct with or without MoSPAB2$^{\Delta SP}$. The SD/-Leu/-Trp/-His medium with 60 mM 3-amino-1,2,4-triazole (3-AT) was used to test for HIS2 expression.

c Activation of the *Bsr-d1* promoter by MoSPAB2 in luciferase assay using *N. benthamiana*. Luciferase signals were imaged (left panel) and measured (right panel) using a dual-LUC assay 48 hr after infiltration. The positive control contains transcription factor EAT1 and *OsLTPL94* promoter driving reporter. EV contains 35S:YFP as the negative control. LUC activities were normalized to REN. Values are mean ± SD, n = 3 samples (one-way ANOVA with Dunnett T3's test, *P* values are shown in the Source Data file). Different letters indicate significant differences ($P < 0.05$). Source data are provided as a Source Data file. Similar results are obtained from three independent biological experiments.

d A schematic diagram of the DNA fragments and probes used for DAP-qPCR and EMSA. The DNA fragments (F1-F9) for DAP-qPCR are indicated by blue lines. The five probes located in F9 used for EMSA are indicated by red lines.

e Binding of MoSPAB1$^{\Delta SP}$ to probe 1 to probe 5 in EMSA. Locations of the probes are as indicated in (D); sequences of the five probes are listed in supplementary table 2. Each probe was labeled with biotin at the 5' end. 6×His was used as a negative control. Similar results are obtained from two independent biological experiments.

f A conserved motif deduced from the probes bound by MoSPAB1 using the MEME suite version 5.4.1. The conserved bases are displayed in colors. M: A/C, Y: C/T.

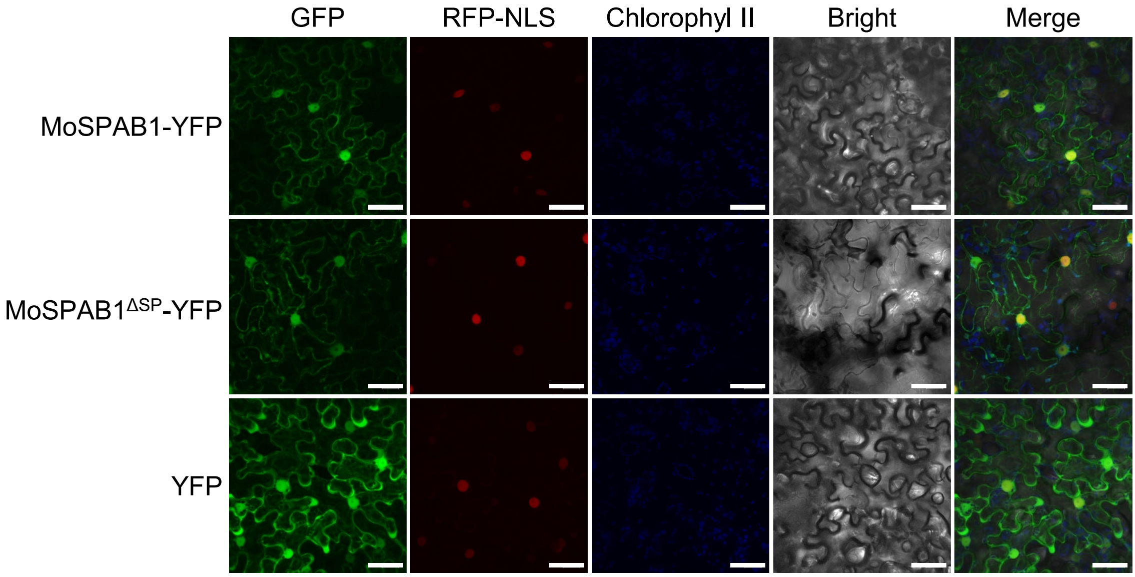

Supplementary Fig. 2 Localization of MoSPAB1-YFP and MoSPAB1$^{\Delta SP}$-YFP in mesophyll cells of *N. benthamiana* leaves. The 35S:MoSPAB1-YFP, 35S:MoSPAB1$^{\Delta SP}$-YFP, and 35S:YFP constructs were used to infiltrate *N. benthamiana* leaves mediated by *Agrobacterium.* Fluorescence images were observed using confocal microscopy 48 hours post inoculation. RFP-NLS serves as a nuclear marker. Chloroplasts are visualized by chlorophyll II, which is represented by blue pseudocolor. Scale bars, 20 μm. Similar results are obtained from two independent biological experiments.

a

MLFTPIRSGL	ASLLALASCA	VAQNV**K**AIYL	F**K**DVMQSDTH	ALKTSGFDTV	50
LLFRIGILPN	ADLVYYSTGS	DGNPVDWPVV	TNGSYVGGQA	LTD**K**ITSL**K**T	100
APTLIERVEV	SLVSHDTTFQ	VI**R**D**R**IAADG	TGASTPLYRA	FDVLKQTWEL	150
DAFNNDDESV	YHVPSTVDFA	QMLGLMGYKY	STAPYTNMNF	WADVQNRINA	200
SVPGLLDRQY	LQVYDGGAAN	NPGTWQT**R**LG	M**K**IVPLLWVN	NDYKPDHGNT	250
PAQAQTRFAN	WNSQYNLAGG	GYWNDYDIEK	LNSSYEGYGG	ALTSVFGQ	298

b

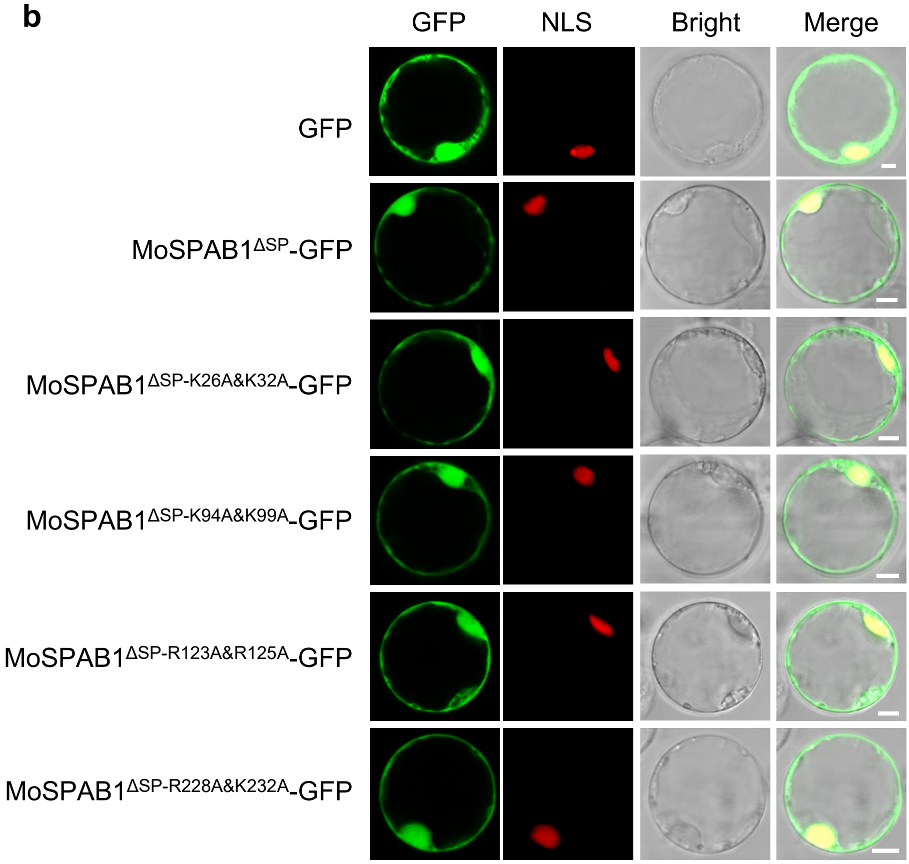

Supplementary Fig. 3 Analysis of nuclear localization signals of MoSPAB1 and localization of mutant MoSPAB1 in rice protoplasts.

a Analysis of nuclear localization signals of MoSPAB1. The underlined amino acids potentially serve as nuclear localization signals. Key amino acids K and R are highlighted in bold.

b Localization of mutant MoSPAB1 in rice protoplasts. The underlined amino acids in bold were changed into alanine (A), individually. MoSPAB1$^{\Delta SP}$-GFP, MoSPAB1$^{K26A\&K32A}$-GFP, MoSPAB1$^{K94A\&K99A}$-GFP, MoSPAB1$^{R123A\&R125A}$-GFP, and MoSPAB1$^{R228A\&K232A}$-GFP were individually expressed transiently in rice protoplasts for 16 hours. Rice protoplasts were examined under a confocal microscope. NLS in PBI221-H2B-mCherry serves as a nuclear marker. Scale bars, 5 μm. Similar results are obtained from two independent biological experiments.

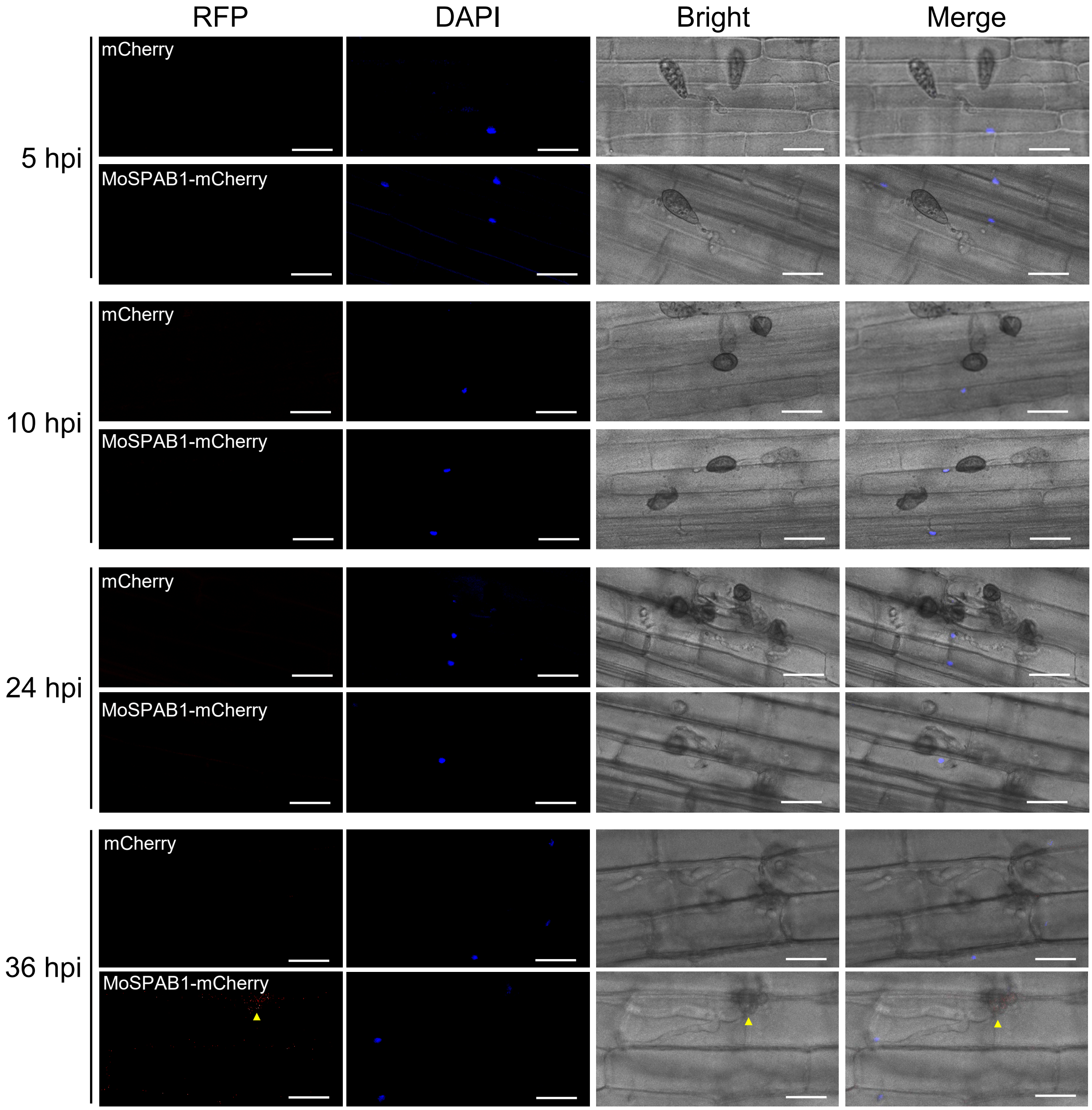

Supplementary Fig. 4 Development of the *MoSPAB1:MoSPAB1-mCherry* strain on rice leaf sheath. Rice leaf sheath was inoculated with conidial suspensions of the *MoSPAB1:MoSPAB1-mCherry* (labeled *MoSPAB1-mCherry*) strain and images taken at 5, 10, 24, and 36 hours post inoculation. *MoSPAB1:mCherry* (labeled mCherry) strain is the negative control. Yellow triangles indicate the MoSPAB1-mCherry in invasion hyphae. Scale bars, 20 μm. Similar results are obtained from two independent biological experiments.

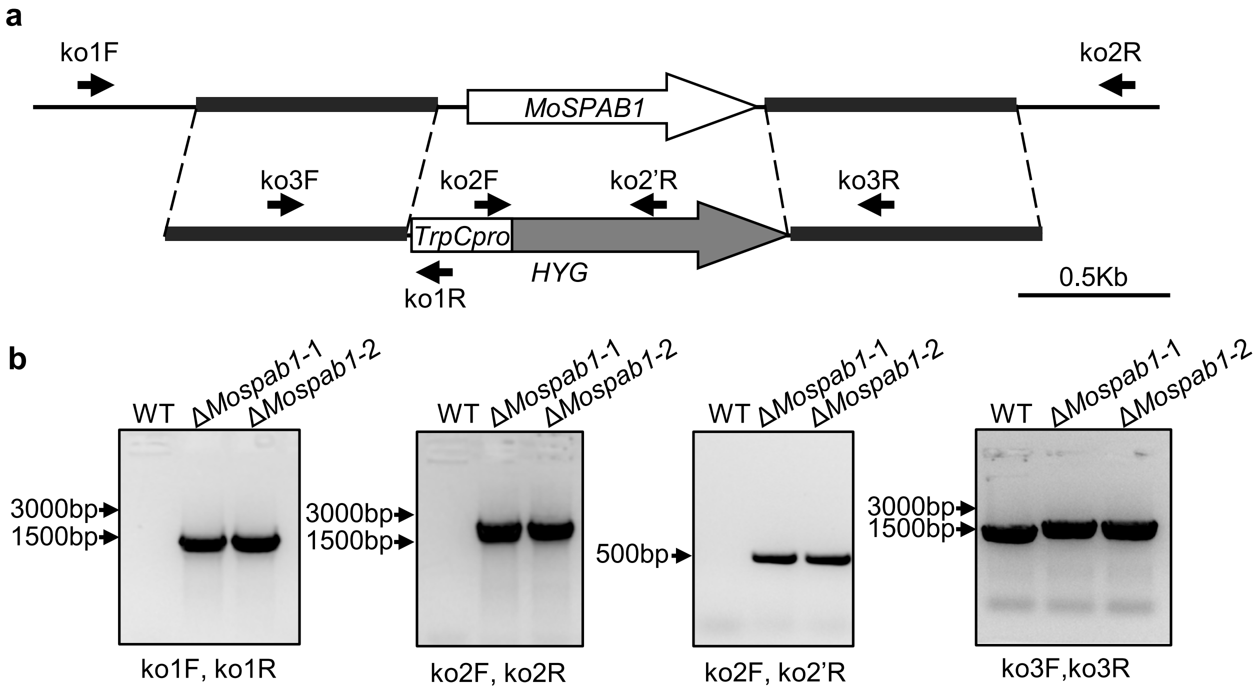

Supplementary Fig. 5 Generation of the Δ*Mospab1* mutant.
a A schematic diagram of targeted deletion of *MoSPAB1* in *M. oryzae*.
b DNA gel blot analysis of the Δ*Mospab1* mutants. Genomic DNA was analyzed by PCR with primer pairs in (a).

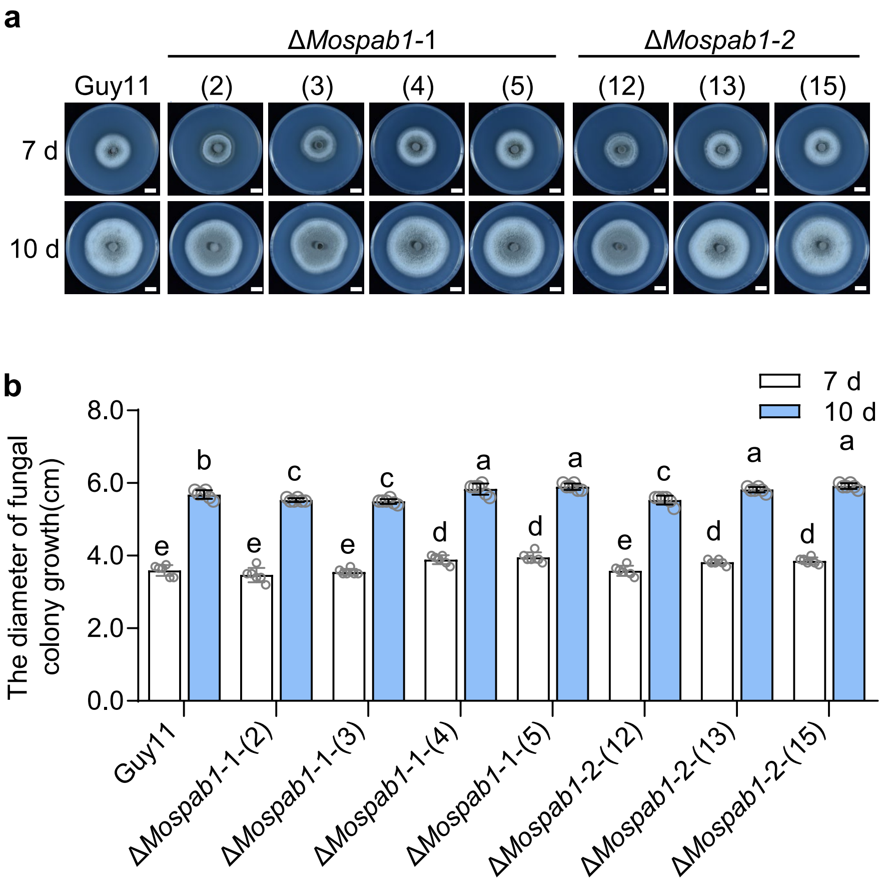

Supplementary Fig. 6 Mycelial growth of Guy11 and the Δ*Mospab1* mutants on complete medium (CM).

a Colonies of Guy11 and the Δ*Mospab1* mutants grown on CM plates for 7 and 10 days. Similar results are obtained from two independent biological experiments.

b Quantified diameters of colonies in (A). Bars represent mean ± SD, n = 6 biologically independent samples (one-way ANOVA with two-sided least significant difference (LSD) test, *P* values are shown in the Source Data file). Different letters indicate significant differences ($P < 0.05$). Source data are provided as a Source Data file.

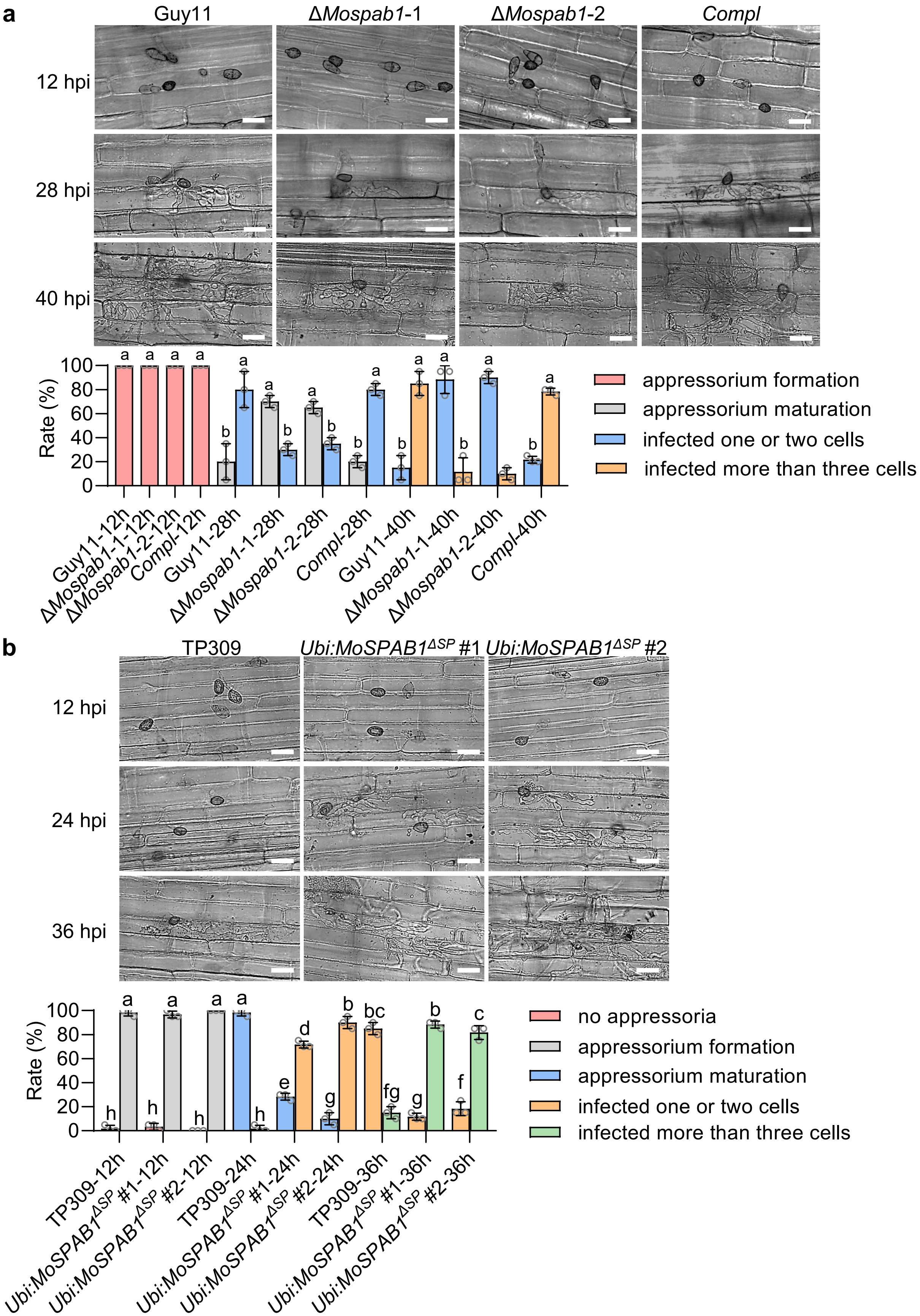

Supplementary Fig. 7 Representative laser-scanning microscopic images of rice sheath cells infected by *M. oryzae*.

a Representative laser-scanning microscopic images of TP309 sheath cells infected by Guy11, Δ*Mospab1*, and a complemented strain individually (upper panel) and distribution of fungal infection progressing at 12, 28, and 40 hpi (lower panel). Scale bars, 20μm. **b** Representative laser scanning microscopic images of TP309 and *Ubi:MoSPAB1*$^{\Delta SP}$ sheath cells infected by Guy11 (upper panel) and distribution of fungal infection progressing at 12, 24, and 36 hpi (lower panel). Scale bars, 20μm. In a and b, a total of 100 spores are counted. n= 3 independent experiments (one-way ANOVA with two-sided least significant difference (LSD) test, *P* values are shown in the Source Data file). Different letters indicate significant differences ($P < 0.05$). Source data are provided as a Source Data file.

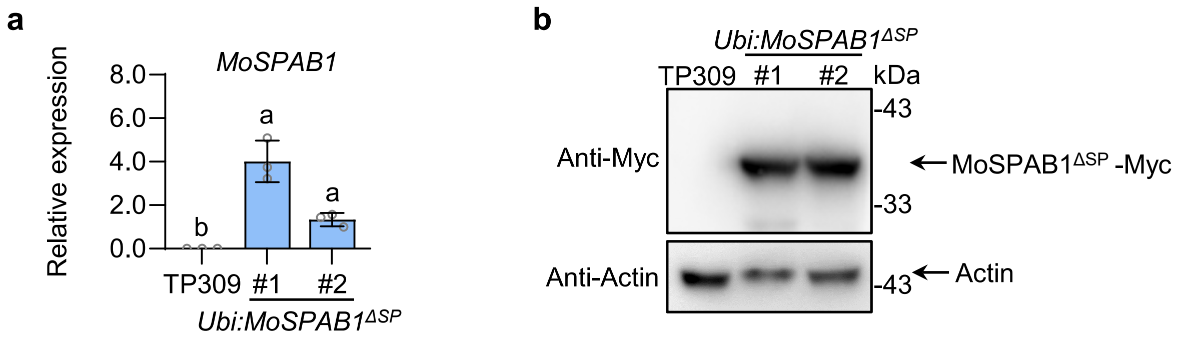

Supplementary Fig. 8 Heterologous *MoSPAB1$^{\Delta SP}$* expression in TP309 rice plants and detection of MoSPAB1 protein.

a *MoSPAB1$^{\Delta SP}$* RNA levels were measured by RT-qPCR. Bars represent mean ± SD (n = 3 biologically independent samples) and analyzed by one-way ANOVA with Dunnett T3's test. *P* values are shown in the Source Data file. Different letters indicate significant differences at $P < 0.05$. Source data are provided as a Source Data file.
b MoSPAB1$^{\Delta SP}$-Myc fusion protein was detected by immunoblotting using an antibody against Myc. Actin was used as a loading control.

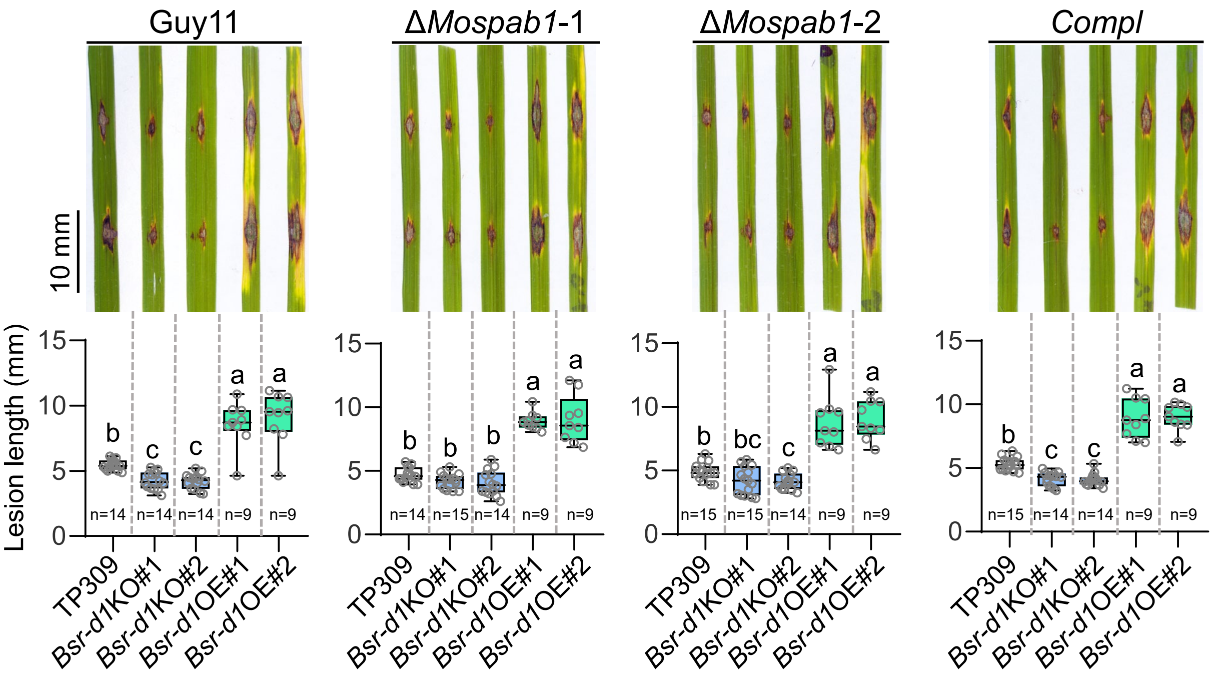

Supplementary Fig. 9 Pathogenicity of Guy11, two Δ*Mospab1* mutants, and a complemented strain on *Bsr-d1* transgenic lines in punch inoculation.

Different *M. oryzae* strains were used to inoculate three-week-old leaves of *Bsr-d1*KO lines, *Bsr-d1*OE lines, and TP309. Photos of leaves with lesions were taken (upper panel) and lesion length quantified (lower panel) 6 dpi. This experiment was done three times. Different letters indicate significant differences at $P < 0.05$. Dunnett T3's multiple-comparison test with one-way ANOVA (mean ± SD; n = 9~15 samples, n is shown in this figure). *P* values are shown in the Source Data file. Similar results are obtained from three independent biological experiments. Source data are provided as a Source Data file.

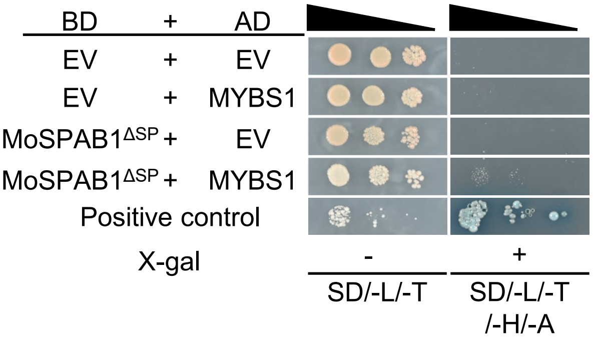

Supplementary Fig. 10 Determination of the MoSPAB1-MYBS1 interaction using the yeast two-hybrid.

Yeast cells containing two plasmids were diluted to 1×, 10×, and 100× with water and inoculated on SD/-L/-T and SD/-L/-T/-H/-A plates with X-gal added and grown at 28 ˚C for 2-4 days. SD/-L/-T represents a medium without leucine and tryptophan. SD/-L/-T/-H/-A represents a medium without leucine, tryptophan, histidine, and adenine. pGBKT7-53 and pGADT7-T were used as the positive control. Similar results are obtained from three independent biological experiments.

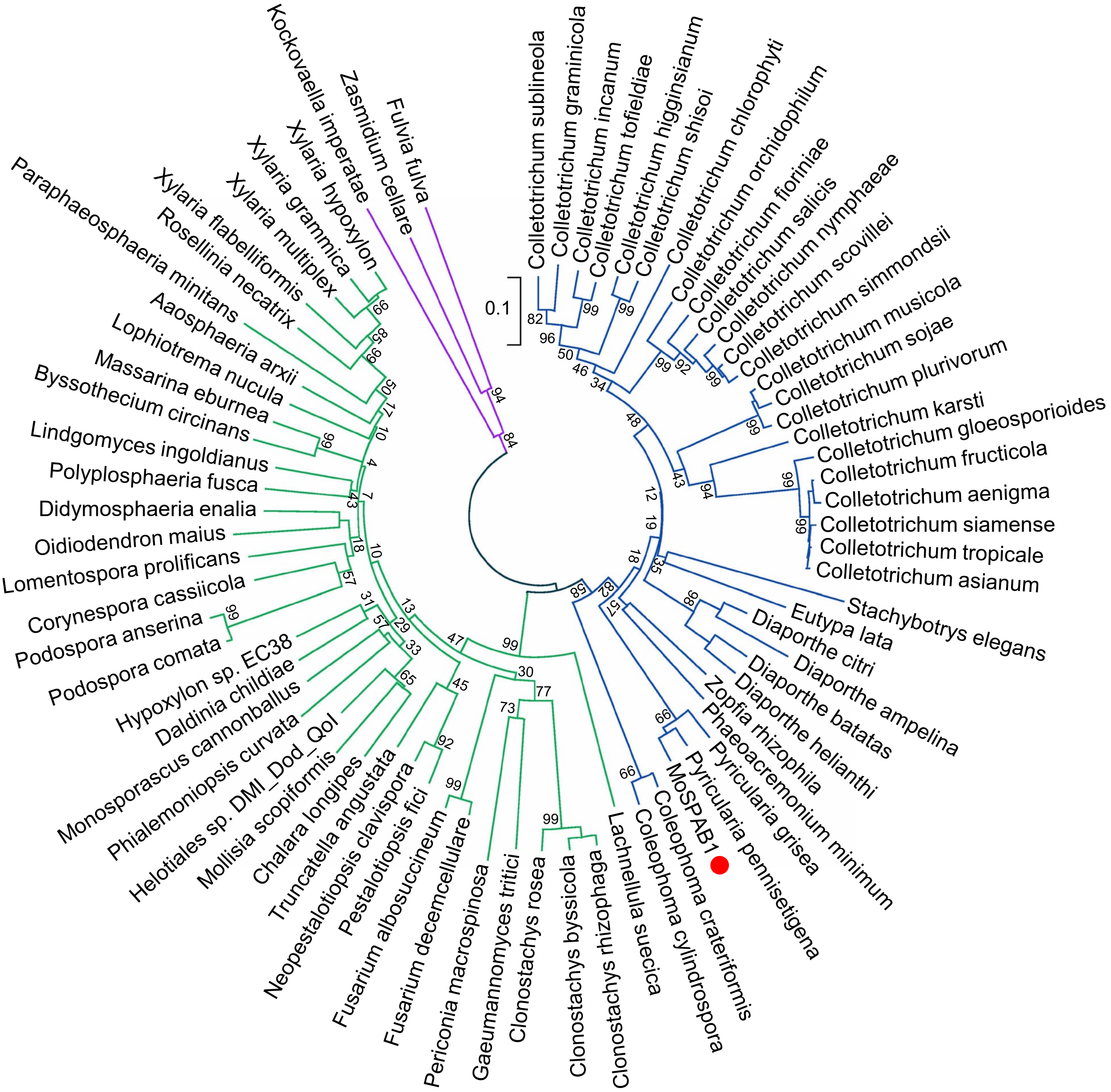

Supplementary Fig. 11 Phylogenetic relationship of MoSPAB1 homologous proteins from fungi. MEGA-X was used to construct the phylogenetic tree. Bootstrap values from 1,000 replicates were used to assess the robustness of the tree.

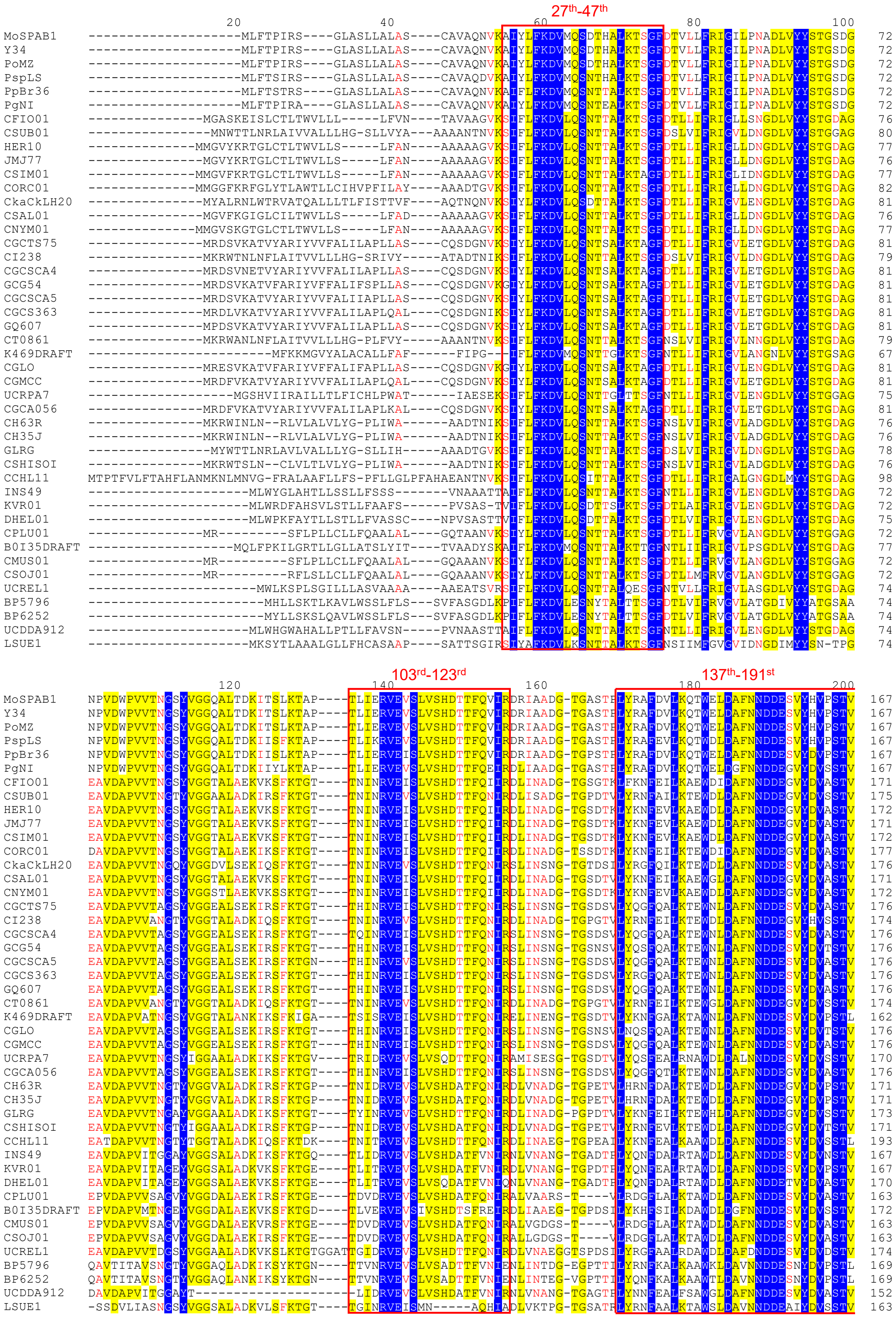

Supplementary Figure 12

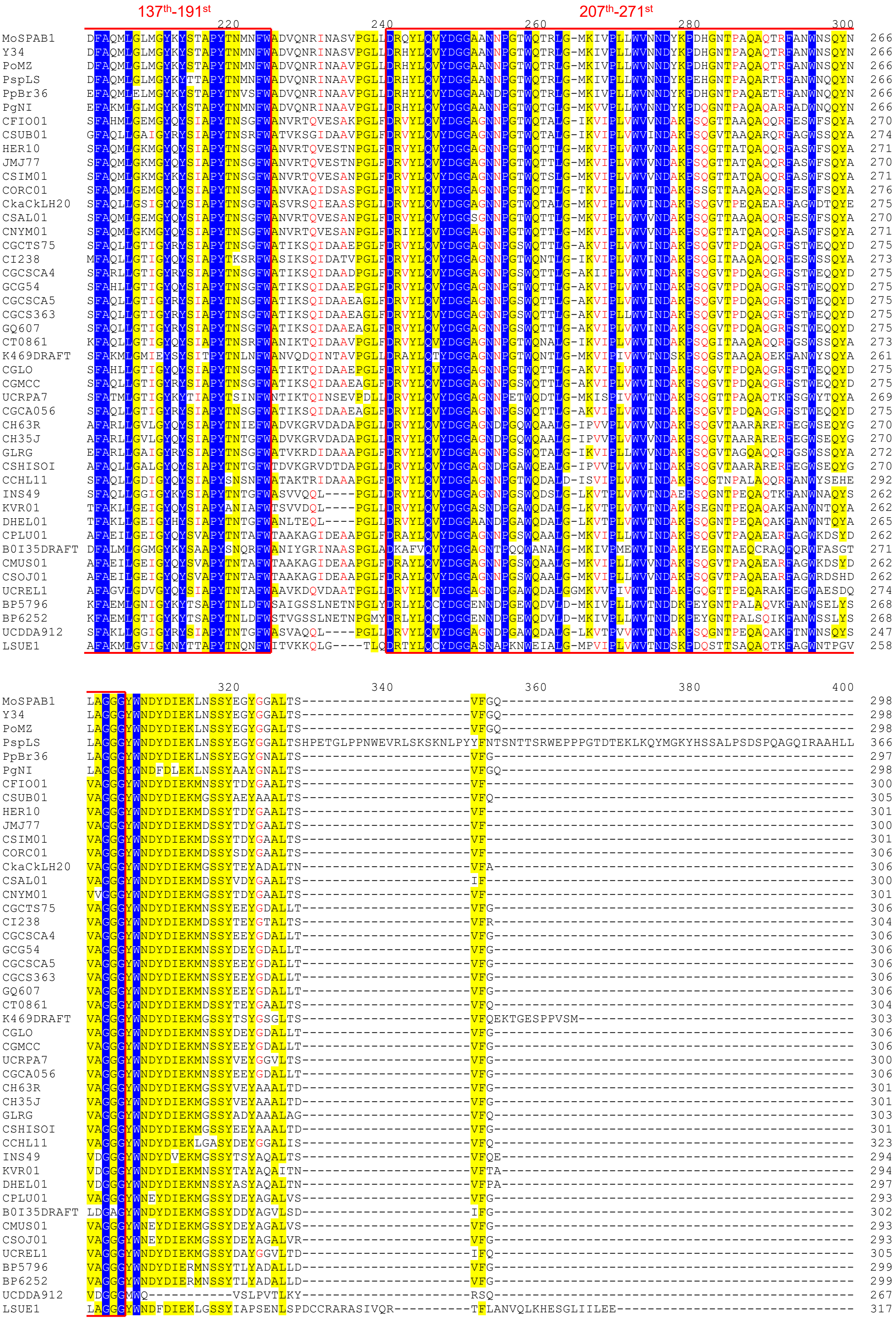

Supplementary Figure 12 continued

Supplementary Fig. 12 Sequence alignment of MoSPAB1 and homologous proteins.
Regions of amino acids 27-47, 103-123, 137-191, and 207-271 were labeled with red boxes. Shades from dark to light indicate similarity degrees of 100%, 80%, 60%, and less than 60%. Y34, the OOU_Y34scaffold01081g from *Pyricularia oryzae* Y34; PoMZ, the PoMZ_02865 from *Pyricularia oryzae*; PspLS, the PspLS_09416 from *Pyricularia* sp. CBS 133598; PpBr36, the PpBr36_02267 from *Pyricularia pennisetigena*; PgNI, the PgNI_02481 from *Pyricularia grisea*; CFIO01, the CFIO01_08786 from *Colletotrichum fioriniae* PJ7; CSUB01, the CSUB01_11810 from *Colletotrichum sublineola*; HER10, the HER10_EVM0008022 from *Colletotrichum scovillei*; JMJ77, the JMJ77_0014689 from *Colletotrichum scovillei*; CSIM01, the CSIM01_07887 from *Colletotrichum simmondsii*; CORC01, the CORC01_09639 from *Colletotrichum orchidophilum*; CkaCkLH20, the CkaCkLH20_04340 from *Colletotrichum karsti*; CSAL01, the CSAL01_01070 from *Colletotrichum salicis*; CNYM01, the CNYM01_12775 from *Colletotrichum nymphaeae* SA-01; CGCTS75, the CGCTS75_v014773 from *Colletotrichum tropicale*; CI238, the CI238_02416 from *Colletotrichum incanum*; CGCSCA4, the CGCSCA4_v013087 from *Colletotrichum siamense*; GCG54, the GCG54_00014612 from *Colletotrichum gloeosporioides*; CGCSCA5, the CGCSCA5_v015001 from *Colletotrichum siamense*; CGCS363, the CGCS363_v013914 from *Colletotrichum siamense*; GQ607, the GQ607_014599 from *Colletotrichum asianum*; CT0861, the CT0861_02089 from *Colletotrichum tofieldiae*; K469DRAFT, the K469DRAFT_741528 from *Zopfia rhizophila CBS 207.26*; CGLO, the CGLO_05580 from *Colletotrichum gloeosporioides* Cg-14; CGMCC, the CGMCC3_g17995 from *Colletotrichum fructicola*; UCRPA7, the UCRPA7_3110 from *Phaeoacremonium minimum* UCRPA7; CGCA056, the CGCA056_v014942 from *Colletotrichum aenigma*; CH63R, the CH63R_12215 from *Colletotrichum higginsianum* IMI 349063; CH35J, the CH35J_009866 from *Colletotrichum higginsianum*; GLRG, the GLRG_09307 from *Colletotrichum graminicola* M1.001; CSHISOI, the CSHISOI_06683 from *Colletotrichum shisoi*; CCHL11, the CCHL11_07208 from *Colletotrichum chlorophyte*; INS49, the INS49_011234 from *Diaporthe citri*; KVR01, the KVR01_009678 from *Diaporthe batatas*; DHEL01, the DHEL01_v204942 from *Diapelianthinianthi*; CPLU01, the CPLU01_12574 from *Colletotrichum plurivorum*; B0I35DRAFT, the B0I35DRAFT_347999 from *Stachybotrys elegans*; CMUS01, the CMUS01_16400 from *Colletotrichum musicola*; CSOJ01, the CSOJ01_1383 from *Colletotrichum sojae*; UCREL1, the UCREL1_11766 from *Eutypa lata* UCREL1; BP5796, the BP5796_12446 from *Coleophoma crateriformis*; BP6252, the BP6252_13955 from *Coleophoma cylindrospora*; UCDDA912, the UCDDA912_g02556 from *Diaporthe ampelina*; LSUE1, the LSUE1_G003910 from *Lachnellula suecica*.

```
Bsr-d1 (+)     -82  AATCATCCACCGCTCGCTGCTGCTGCTA  -55
                     |     ||||||    ||  ||
SbBsr-d1 (+)  -809  TGCACACAGTAGCTCGCTCGCGCACCTT  -782

Bsr-d1 (+)     -82  AATCATCCACCGCTCGC-TGCTGCTGCTA  -55
                      |   |||  |      || |    |
AcBsr-d1 (-)  -159  GCTACATGAATGCTTGTACACTACATAT-  -186

Motif               (C/A)(G/C)(C/G)T(T/C)G(C/A)(T/C）
```

Supplementary Fig. 13 Conserved cis-motif (C/A)(G/C)(C/G)T(T/C)G(C/A)T bound by MoSPAB1 was found in *SbBsr-d1* and *AcBsr-d1* promoters.
'+' and '-' behind the gene represent the sense and antisense strand, respectively.
