# Supplementary material for: Magnaporthe oryzae effector MoSPAB1 directly activates rice Bsr-d1 expression to facilitate pathogenesis
Source: Nat Commun. 2023 Dec 18;14:8399. doi: 10.1038/s41467-023-44197-9 (PMC10728069; doi:10.1038/s41467-023-44197-9)
Supplement: Supplementary file 3 — Description of Additional Supplementary Files [file 41467_2023_44197_MOESM3_ESM.pdf]

Description of Additional Supplementary Files

Supplementary Data 1. Primers used in this study
