# Supplementary material for: Magnaporthe oryzae effector MoSPAB1 directly activates rice Bsr-d1 expression to facilitate pathogenesis
Source: Nat Commun. 2023 Dec 18;14:8399. doi: 10.1038/s41467-023-44197-9 (PMC10728069; doi:10.1038/s41467-023-44197-9)
Supplement: Supplementary file 5 — Reporting Summary [file 41467_2023_44197_MOESM5_ESM.pdf]

nature portfolio

Corresponding author(s): Xuewei Chen and Weitao Li

Last updated by author(s): Dec 4, 2023

Reporting Summary

Nature Portfolio wishes to improve the reproducibility of the work that we publish. This form provides structure for consistency and transparency in reporting. For further information on Nature Portfolio policies, see our Editorial Policies and the Editorial Policy Checklist.

Statistics

For all statistical analyses, confirm that the following items are present in the figure legend, table legend, main text, or Methods section.

n/a	Confirmed	
☐	☒	The exact sample size (*n*) for each experimental group/condition, given as a discrete number and unit of measurement
☐	☒	A statement on whether measurements were taken from distinct samples or whether the same sample was measured repeatedly
☐	☒	The statistical test(s) used AND whether they are one- or two-sided<br>*Only common tests should be described solely by name; describe more complex techniques in the Methods section.*
☒	☐	A description of all covariates tested
☐	☒	A description of any assumptions or corrections, such as tests of normality and adjustment for multiple comparisons
☐	☒	A full description of the statistical parameters including central tendency (e.g. means) or other basic estimates (e.g. regression coefficient) AND variation (e.g. standard deviation) or associated estimates of uncertainty (e.g. confidence intervals)
☐	☒	For null hypothesis testing, the test statistic (e.g. *F*, *t*, *r*) with confidence intervals, effect sizes, degrees of freedom and *P* value noted<br>*Give P values as exact values whenever suitable.*
☒	☐	For Bayesian analysis, information on the choice of priors and Markov chain Monte Carlo settings
☒	☐	For hierarchical and complex designs, identification of the appropriate level for tests and full reporting of outcomes
☒	☐	Estimates of effect sizes (e.g. Cohen's *d*, Pearson's *r*), indicating how they were calculated

Our web collection on statistics for biologists contains articles on many of the points above.

Software and code

Policy information about availability of computer code

Data collection	Leica LAS X Hardware Configurator Version 2020.6.0 (Leica Microsystems CMS GmbH) was used to collect the micrographs. Image Lab version 3.0 build 11 (Bio-Rad) was used for DNA electrophoresis and WB data collection.
Data analysis	MEGA X was used for sequence alignment; SPSS 27.0 was used for data analysis; GraphPad Prism 8 was used for making pictures; MEME Suite 5.4.1 was used for analyzing conserved motifs; SignalP 5.0 was used for analyzing the signal peptide.

For manuscripts utilizing custom algorithms or software that are central to the research but not yet described in published literature, software must be made available to editors and reviewers. We strongly encourage code deposition in a community repository (e.g. GitHub). See the Nature Portfolio guidelines for submitting code & software for further information.

Data

Policy information about availability of data

All manuscripts must include a data availability statement. This statement should provide the following information, where applicable:

- Accession codes, unique identifiers, or web links for publicly available datasets
- A description of any restrictions on data availability
- For clinical datasets or third party data, please ensure that the statement adheres to our policy

The data including MGG_07390, MGG_08499, LOC_Os03g32230, LOC_Os01g34060, SORBI_3001G330100, CEY00_Acc21054, CSUB01_11810, CGMCC3_g17995, and et al are opened and availabile, and there are no restrictions on data availabilty. For example, MGG_07390 and MGG_08499 are from the EndemblFungi database [https://fungi.ensembl.org/Magnaporthe_oryzae/Info/Index]; SORBI_3001G330100 (XP_002465089.1), CSUB01_11810 (KDN66937), and

CGMCC3_g17995 (XP_031875374.1) from the National Center for Biotechnology Information database [https://www.ncbi.nlm.nih.gov/]; LOC_Os03g32230, LOC_Os01g34060, and LOC_Os01g73170 from the Rice Genome Annotation Project database [http://rice.uga.edu/]; CEY00_Acc21054 from the Gramene database [https://www.gramene.org/].

Research involving human participants, their data, or biological material

Policy information about studies with human participants or human data. See also policy information about sex, gender (identity/presentation), and sexual orientation and race, ethnicity and racism.

Reporting on sex and gender	No data
Reporting on race, ethnicity, or other socially relevant groupings	No data
Population characteristics	See above
Recruitment	No recruitment required
Ethics oversight	No ethics oversight required

Note that full information on the approval of the study protocol must also be provided in the manuscript.

Field-specific reporting

Please select the one below that is the best fit for your research. If you are not sure, read the appropriate sections before making your selection.

☒ Life sciences ☐ Behavioural & social sciences ☐ Ecological, evolutionary & environmental sciences

For a reference copy of the document with all sections, see nature.com/documents/nr-reporting-summary-flat.pdf

Life sciences study design

All studies must disclose on these points even when the disclosure is negative.

Sample size	Based on the usual calculation method, at lest three samples were chosen. Duncan's multiple-comparison test with one-way ANOVA and two-sided Student's t-test were used.
Data exclusions	No data were excluded from the analyses
Replication	In a parallel experiment, we set up triple replications for determining the mean value and standard deviation. All attempts ar replication were successful.
Randomization	Allocation was random
Blinding	Blind testing was not routinely carried out in this study because it was not relevant to most of the experiments carried out and these were based on molecular genetic and biochemical characterizayion of MoSPAB1, thus requiring knowledge of samples to be processed.

Reporting for specific materials, systems and methods

We require information from authors about some types of materials, experimental systems and methods used in many studies. Here, indicate whether each material, system or method listed is relevant to your study. If you are not sure if a list item applies to your research, read the appropriate section before selecting a response.

Materials & experimental systems

n/a	Involved in the study	
☐	☒	Antibodies
☒	☐	Eukaryotic cell lines
☒	☐	Palaeontology and archaeology
☒	☐	Animals and other organisms
☒	☐	Clinical data
☒	☐	Dual use research of concern
☐	☒	Plants

Methods

n/a	Involved in the study	
☒	☐	ChIP-seq
☒	☐	Flow cytometry
☒	☐	MRI-based neuroimaging

Antibodies

Antibodies used	Cell Signaling Technology, Cat#2276S, Myc-Tag (9B11) Mouse mAb, Lot#24; Easybio, Cat#BE0102, Goat Anti-Mouse IgG (H&L)-HRP Conjugated for Myc, Lot#80781104; Sangon Biotech, Cat#D110007, Anti-ACTIN (Plants) rabbit polyclonal antibody, Lot#GC23AA006; Easybio, Cat#BE0101, Goat Anti-Rabbit IgG (H&L)-HRP Conjugated for ACTIN, Lot#80870217; Sangon Biotech, Cat# D191001, Anti-6×His Tag mouse monoclonal antibody, Lot#1606AA005A; Sangon Biotech, Cat# D110271, Anti-GST Tag rabbit polyclonal antibody, Lot#1919AA008.
Validation	The anti-His and anti-GST antibodies were used in pulldown assay for detection of His-MoSPRB1ΔSP and GST-MYBS1, respectively. The anti-Myc and anti-ACTIN antibodies were used in Western blot on rice expressing MoSPRB1ΔSP-Myc. Validation statements are on the manufacturer's website.

Dual use research of concern

Policy information about dual use research of concern

Hazards

Could the accidental, deliberate or reckless misuse of agents or technologies generated in the work, or the application of information presented in the manuscript, pose a threat to:

No	Yes	
☒	☐	Public health
☒	☐	National security
☒	☐	Crops and/or livestock
☒	☐	Ecosystems
☒	☐	Any other significant area

Experiments of concern

Does the work involve any of these experiments of concern:

No	Yes	
☒	☐	Demonstrate how to render a vaccine ineffective
☒	☐	Confer resistance to therapeutically useful antibiotics or antiviral agents
☒	☐	Enhance the virulence of a pathogen or render a nonpathogen virulent
☒	☐	Increase transmissibility of a pathogen
☒	☐	Alter the host range of a pathogen
☒	☐	Enable evasion of diagnostic/detection modalities
☒	☐	Enable the weaponization of a biological agent or toxin
☒	☐	Any other potentially harmful combination of experiments and agents
