# Supplementary material for: Magnaporthe oryzae effector MoSPAB1 directly activates rice Bsr-d1 expression to facilitate pathogenesis
Source: Nat Commun. 2023 Dec 18;14:8399. doi: 10.1038/s41467-023-44197-9 (PMC10728069; doi:10.1038/s41467-023-44197-9)
Supplement: Supplementary file 6 — Source Data [file 41467_2023_44197_MOESM6_ESM.zip › uncropped blots.pdf]

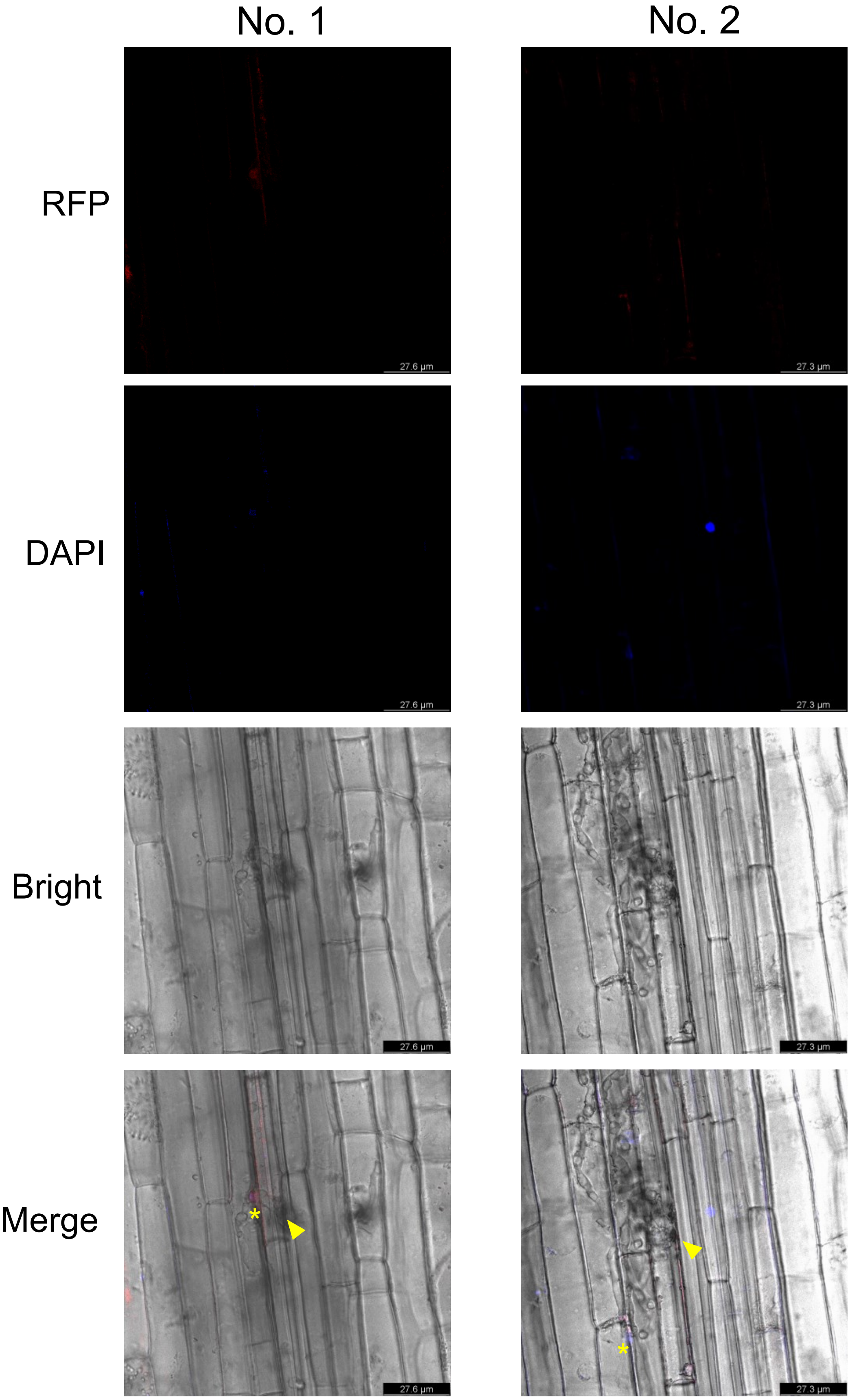

Additional images of localization of MoSPAB1-mCherry in rice sheath cells 48 hours post inoculation.
The upper panel shows fungal cells labelled with mCherry using the *MoSPAB1* promoter to express the protein. DAPI was used to stain the nuclei. Yellow asterisks indicate the presence of MoSPAB1-mCherry in the rice nucleus. Yellow triangles represent the appressorium.

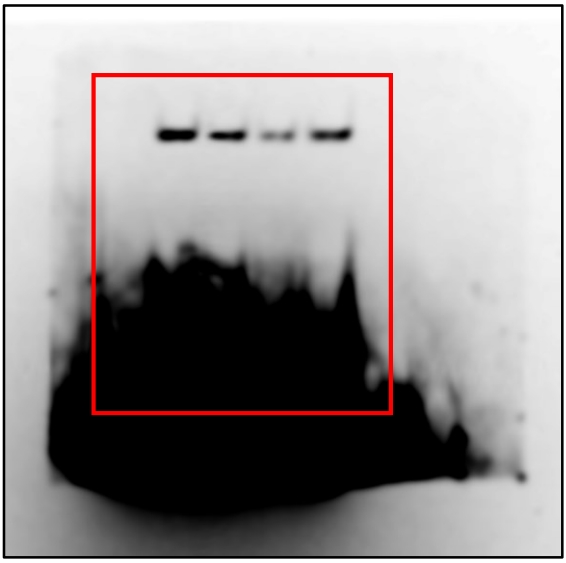

Figure 1f

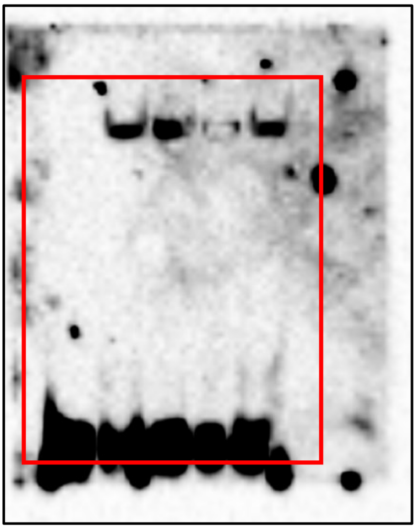

Figure 1g

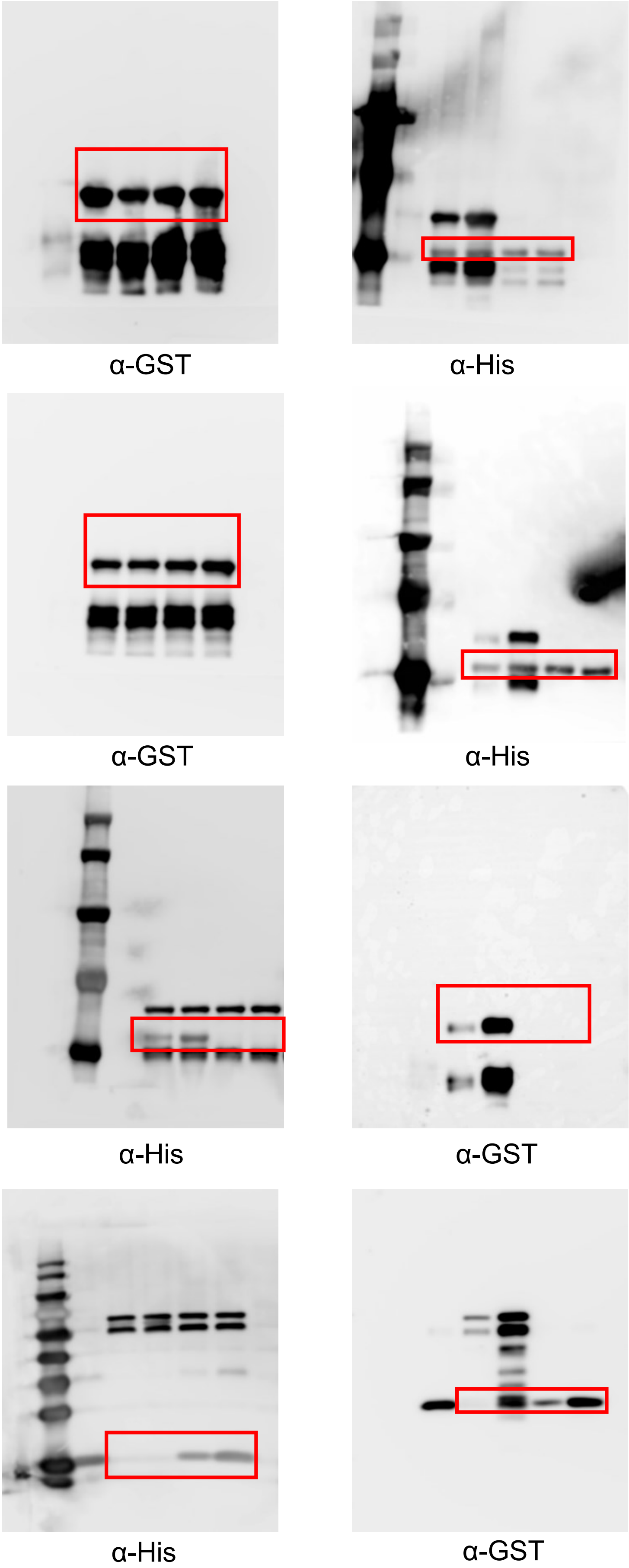

Figure 3f

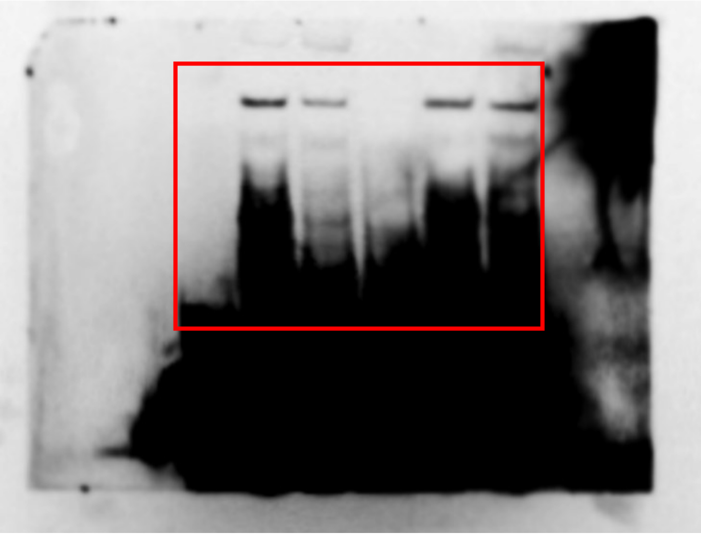

Figure 4a

Supplementary data

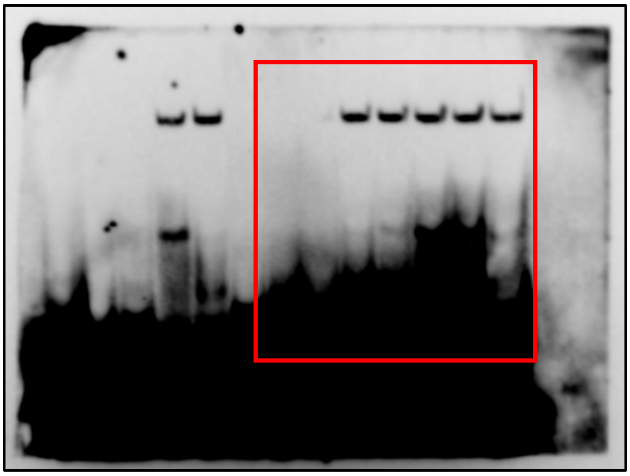

Supplementary Figure 1e

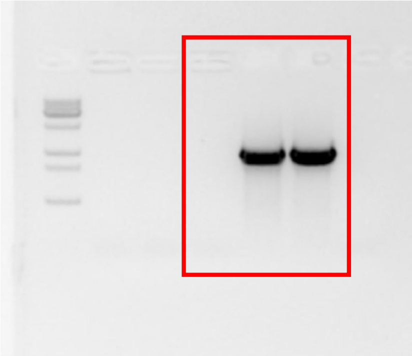

ko1F, ko1R

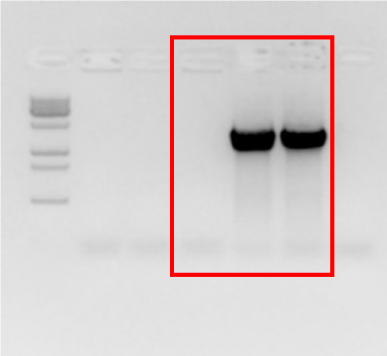

ko2F, ko2R

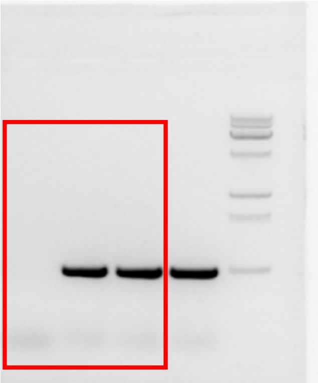

ko2F, ko2’R

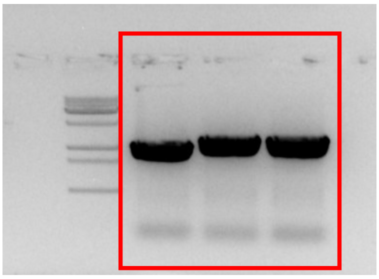

ko3F, ko3R

Supplementary Figure 5b

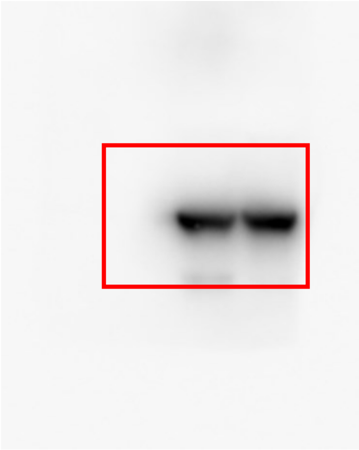

Anti-Myc

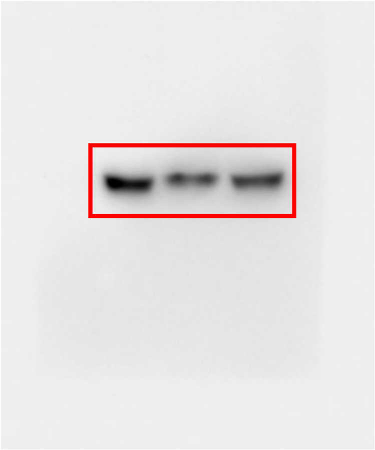

Anti-Actin

Supplementary Figure 8b
